# A Critical Review on Polyphenols and Health Benefits of Black Soybeans

**DOI:** 10.3390/nu9050455

**Published:** 2017-05-04

**Authors:** Kumar Ganesan, Baojun Xu

**Affiliations:** Food Science and Technology Program, Beijing Normal University-Hong Kong Baptist University United International College, Zhuhai 519085, China; kumarganesan@uic.edu.hk

**Keywords:** polyphenols, black soybean, antioxidants, human diseases, health benefits

## Abstract

Polyphenols are plant secondary metabolites containing antioxidant properties, which help to protect chronic diseases from free radical damage. Dietary polyphenols are the subject of enhancing scientific interest due to their possible beneficial effects on human health. In the last two decades, there has been more interest in the potential health benefits of dietary polyphenols as antioxidant. Black soybeans (*Glycine max* L. Merr) are merely a black variety of soybean containing a variety of phytochemicals. These phytochemicals in black soybean (BSB) are potentially effective in human health, including cancer, diabetes, cardiovascular diseases, cerebrovascular diseases, and neurodegenerative diseases. Taking into account exploratory study, the present review aims to provide up-to-date data on health benefit of BSB, which helps to explore their therapeutic values for future clinical settings. All data of in vitro and in vivo studies of BSB and its impact on human health were collected from a library database and electronic search (Science Direct, PubMed, and Google Scholar). The different pharmacological information was gathered and orchestrated in a suitable spot on the paper.

## 1. Polyphenols

Polyphenols are phytochemicals, found largely in fruits, vegetables, tea, coffee, chocolates, legumes, cereals, and beverages. There are over 8000 polyphenols identified in nature and their main functions are as antioxidant. They protect our body from free radical damage and defense against UV radiation or aggression by pathogens. In the last two decades, there has been more interest in the potential health benefits of dietary polyphenols as antioxidant. The average 100 grams fresh weight of fruits (grapes, apple, pear, cherries, and berries) contain up to 300 mg of polyphenols. Typically, a cup of tea or coffee or a glass of red wine contains more than 100 mg of polyphenols. In addition, cereals, vegetables, dry legumes and chocolate also contribute to the polyphenolic intake and thereby protect our body from chronic diseases [1]. Dietary polyphenols are the subject of enhancing scientific interest due to their possible beneficial effects on human health. They are usually provided to the food as color, flavor, bitter, and astringent, and maintain stability from oxidation. Several epidemiological studies and associated meta-analyses strongly showed that the consumption of these polyphenols offered better protection against chronic diseases such as cancers, cardiovascular diseases, cerebrovascular diseases, diabetes, ageing and neurodegenerative diseases [2,3,4,5].

### 1.1. Types of Polyphenols

Polyphenols are divided into four different categories based on the presence of number of phenolic groups and structural elements [6]. Food usually contains complex polyphenols, predominantly found in the outer layers of the plants [1].

1. Flavonoids: Have a potential effect on radical scavenging and inflammatory reactions. They are predominantly found in fruits, vegetables, legumes, red wine, and green tea. They are further divided into a number of subgroups namely, flavones, flavonols, flavanones, isoflavones, anthocyanidins, chalcones, and catechins.
2. Stilbenes: Found in product of graphs, red wine, and peanuts. Resveratrol is the most well-known compound among the group.
3. Lignans: Found in seeds like flax, linseed, legumes, cereals, grains, fruits, algae, and certain vegetables.
4. Phenolic acids: Found in coffee, tea, cinnamon, blueberries, kiwis, plums, apples, and cherries and have two subgroups, namely hydroxybenzoic acids, and hydroxycinnamic acids.

### 1.2. Role of Polyphenols in Plants and Humans

**Figure 1:**
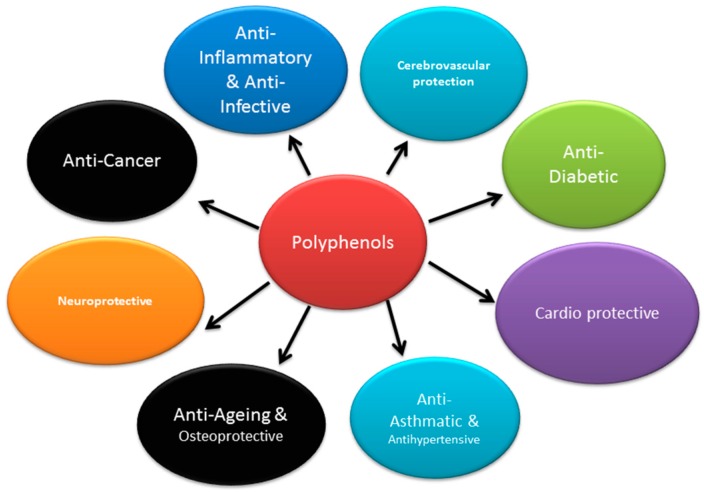
Role of polyphenols in humans.

In the plant, polyphenols protect from UV radiation, pathogens, oxidative stress, and harsh climatic conditions [1]. In the human body, polyphenols are antioxidants, and have diverse biological properties such as anti-diabetic [7,8], anticancer [9,10], anti-inflammatory [11,12], cardioprotective [13], osteoprotective [14,15], neuroprotective [16,17], antiasthmatic [18], antihypertensive [19], antiageing [20], antiseptic [21], cerebrovascular protection [22], cholesterol lowering [23], hepatoprotective [24], antifungal [25], antibacterial [26] and antiviral properties [27] (Figure 1).

## 2. Black Soybeans

Soybeans contain various colors of seed coat including black, yellow, green, and brown. It is due to the presence of anthocyanins, chlorophyll, and other pigments. Black soybeans (BSB) have been widely consumed as food and as material for Oriental medicine for hundreds of years in Asia. The black pigmentation is due to accumulation of anthocyanins in the epidermis palisade layer of the seed coat [28]. Different anthocyanins in BSB have been identified, including cyanidin-3-glucoside, delphinidin-3-glucoside, and pelargonidin-3-Glucoside [29]. BSB is an excellent dietary source for disease prevention and health promotion.

In the last two decades, isoflavones and proteins are the primary health beneficial components in BSB that have received attention [30,31,32]. Nevertheless, there are insufficient data to explain the health benefits exclusive to BSB. They have potentially active phytochemicals such as isoflavones, sterols, phytic acid, saponins, and phenolics, which are potentially effective for human health and prevention of various chronic diseases [32]. Research showed that BSB has the greatest antioxidant properties compared to other colored soybeans [33,34]. The characteristic antioxidant potential is due the presence of phenolics, which is mainly distributed in the seed coat [35,36]. In the seed coat, around 20 phenolic compounds, predominantly six anthocyanins, are greatly (13–50 times) distributed in several BSB varieties [32], which helps to reduce the risk of chronic diseases such as metabolic disorders and cancers [37,38,39,40]. These varieties have the potential to be used in functional foods and food colorant development. The predominant quantity of anthocyanins provides a black color to the seed coat and showed to have potent antioxidant properties, which is mainly responsible for health-promoting effects of BSB. In addition to the anthocyanins, BSB contain other phenolics such as tannins, isoflavones and phenolic acids [34,35].

### Nutritional Importance of BSB

BSB has a high content of protein (32–43.6%). In addition to protein, BSB contains carbohydrates (31.7–31.85%), lipids (15.5–24.7%), water (5.6–11.5%), minerals (calcium, phosphorous, magnesium, potassium, sodium, selenium, etc.) and vitamins (Vitamin E, B complex, etc.) [41,42]. The BSB lipid composition consists of 86% unsaturated fatty acids, especially linoleic (6.48–11.6%), linolenic (0.72–2.16%) and oleic acids (3.15–8.82%), making it beneficial to human health [43]. Soybean is characterized by the most digestible proteins, lysine and methionine. However, it is limited by sulfur amino acids and tryptophan.

## 3. Anthocyanins Rich BSB

**Figure 2:**
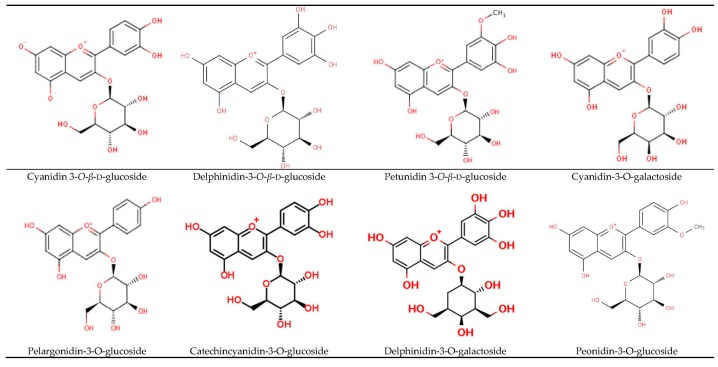
Anthocynins isolated from back soybean [45,46,47].

Anthocyanins are water-soluble natural pigments that belong to flavonoids, a larger subgroup of polyphenols, and widely distributed in BSB and shown to provide numerous health benefits. Major anthocyanins have been isolated and identified from the seed coat of BSB such as cyanidin 3-*O*-*β*-d-glucoside, delphinidin-3-*O*-*β*-d-glucoside, pelargonidin-3-*O*-glucoside, and petunidin 3-*O*-*β*-d-glucoside [29,44]. The minor anthocyanins such as catechincyanidin-3-*O*-glucoside, delphinidin-3-*O*-galactoside, cyanidin-3-*O*-galactoside, and peonidin-3-*O*-glucoside have also been isolated and identified based on the fragmentation patterns of high-performance liquid chromatography–diode array detector–electrospray ionization/mass spectrometry analysis [45,46]. 5,7,3′,4′-Tetrahydroxyflav-2-en-3-ol 3-*O*-*β*-d-glucoside have also been isolated from immature BSB [47]. The structure of anthocyanins-rich BSB is depicted in Figure 2.

## 4. Health Benefits of Anthocyanins Rich BSB

Anthocyanins rich BSB has potential health benefit as complementary medicine and utilized in various formulation implied for antioxidant, anti-inflammatory, nephroprotective, antidiabetic, anticancer, anti-infertility, anti-obesity, anti-arthritic, neuroprotective, antihyperlipidemic, anti-cataract and wound healing properties. Detailed information on dose range, route of administration, the model used, negative controls, and other pharmacological results based on the experimental research study in vivo and in vitro, according to the appropriate title depicted, are presented in Table 1.

Some of the health benefits of anthocyanins rich BSB are listed below.

### 4.1. Enhance Bone Stability

BSB has high content of proteins and fibers. It has an enormous amount of minerals such as calcium, phosphorous magnesium, iron, manganese, copper, and zinc, which contribute to maintain and stabilize the bone and its strength [99,100]. The study showed that the consumption of BSB has a definite protective effect on bone loss in postmenopausal osteoporosis study model and thereby BSB inhibits bone turnover and prevent bone resorption. This study confirmed that the intake of BSB can be used to prevent bone loss in estrogen deficiency animal studies [101]. In addition, the observational and acute clinical trial studies also suggested that the isoflavone-genistein reduces bone loss and enhances bone mineral density in osteopenic postmenopausal women [102].

### 4.2. Reduce Blood Pressure

Owing to the beneficial health effects on the human cardiovascular system, BSB has been a focus of intensive research. BSB contains a low concentration of sodium, which helps to maintain blood pressure at a normal range. A recent epidemiological study also showed that the anthocyanins rich BSB reduce the risk of cardiovascular diseases and maintain the blood pressure in the affected individuals [38,103]. In addition, BSB has potent inhibitory activity on collagen-induced platelet aggregation and reduce cardiovascular risk, and thereby improves blood circulation [104]. BSB has enormous quantities of fiber, potassium, folic acid, pyridoxal phosphate, and phytonutrients (quercetin and saponins) and lack of cholesterol, which supports to reduce cardiovascular complications. The fiber in BSB helps to lower total cholesterol (TC), LDL-cholesterol (LDL-C) in the blood and liver that reduce the risk of heart disease. It inhibits oxidative stress in postmenopausal women by increasing antioxidant activity and improving lipid profiles [105].

### 4.3. Reduce Cardiovascular Complications

The consumption of BSB may reduce the risk of coronary heart diseases. Recent researches have shown that BSB inhibit the effect of low density lipoprotein oxidation, inhibit TNF-alpha-induced vascular cell adhesion molecule-1 (VCAM), intracellular adhesion molecule-1 (ICAM), and cyclooxygenase-2 levels [83]. Further, anthocyanins protected myocardial injury from ischemia-reperfusion in rats [83]. Thus, anthocyanins from BSB seed coat benefit for pathological conditions like coronary heart diseases [84].

### 4.4. In Managing Diabetes

BSB is known to be rich in anthocyanins, and they have been consumed since ancient times for their beneficial effects on health. It has been reported that BSB seed coat may ameliorate obesity and insulin resistance [79]. BSB has more fibers, which have a vital role to reduce the blood sugar. Notably, one cup of BSB contributes around 15 g of fibers [106]. In addition, the BSB seed coat extract contains polyphenol-rich food material consisting of 9.2% cyanidin 3-glucoside, 6.2% catechins, 39.8% procyanidins, and others. These compounds remarkably prevent obesity and diabetes by enhancing energy expenditure and suppressing inflammation [107,108].

### 4.5. Cancer Prevention

Several studies showed that anthocyanins rich BSB have been shown to inhibit cancer cell growth by suppressing oxidative stress and inflammatory responses. Anthocyanin-rich BSB seed coat extract possibly reduces the development of tumors in various organs such as intestines [51], breast [109], prostate [65,110], stomach [80], ovary [111], endometrium [112], and liver [113]. In addition to the anthocyanins, saponins also prevent cancer cells from proliferation and spreading throughout the body [114]. BSB is high in folic acid, which plays a vital role in DNA synthesis and repair, thus BSB prevents the formation of cancer cells from DNA mutations [115].

### 4.6. Reduce Body Weight

BSB contain high quantity of fibers, which enhance satiety and decrease appetite, making an individual feel full for longer time, and thereby reduce the overall calorie intake [116]. Many studies have suggested that the consumption of anthocyanins rich BSB reduces the risk of adipogenic activity, obesity [40,117], fatty acid content in subcutaneous [118] and overall mortality while promoting a healthy complexion, visceral fat, increased energy, and overall reduce the body weight [49].

### 4.7. Antimicrobial Actions

Anthocyanins rich BSB may have antibacterial, anti-fungal, and anti-viral properties. The extract from BSB produced significant growth reductions of food borne pathogens such as *Escherichia coli, Salmonella typhimurium* and *Campylobacter jejuni* in broth-cultures as well as on chicken skin [119]. A previous study also showed an isolated monomeric protein (molecular mass of 25 kDa), containing N-terminal sequence, which resembles a segment of chitin synthase. The protein, named glysojanin, demonstrated a potent antifungal activity against *Fusarium oxysporum* and *Mycosphaerella arachidicola* and antiviral potential against HIV-1, human adenovirus type 1 and coxsackievirus B1 [98,120].

## 5. Conclusions

Black soybeans have popularly been utilized as a food and medicinal material for a long time with low price. Anthocyanins have antioxidant effect and can be useful for the treatment of diabetes, cardiovascular disorders, cancers, etc. Although the exact mechanism by which anthocyanins prevent the expression of adhesion molecules remains to be elucidated, they can be used as good materials to modulate or prevent such chronic diseases. In any case, more support for such properties/dynamic constituents has been acquired from cellular and molecular studies, while clinical studies are as yet inadequate. Since animal research does not generally interpret human circumstances, additional clinical studies are justified for comprehending the full interpretation of the effects of Anthocyanins in BSB for human disease prevention. Subsequently, futures far-reaching clinical studies are required to warrant the therapeutic convenience of anthocyanins in BSB. Furthermore, highlighting the synergistic multi-component effects of BSB on biological functions would be a recommendation for further studies, as well as studies of the mechanism of action and new biomarkers to prove the effectiveness of BSB bioactive compounds in preventing and treating several symptoms and/or pathologies.

## Figures and Tables

**Table 1:** Summary of in vivo and in vitro studies of health benefit potentials of Anthocyninsrich BSB.

Model	Anthocyanin Rich BSB	Dose and Route of Administration	Negative Control	Investigation	Results	Reference
Mouse	Anthocyanin	24 mg/kg/day PO	Lipopolysaccharide	Assay of phospho-c-JNK1, IL-1β, TNF-α, transcription factor NF-κB, GFAP, Iba-1, Bax, cytosolic cytochrome C, cleaved caspase-3 and PARP-1	Neuroprotective activity	[48]
Obesity human	Anthocyanin-rich BSB testa extracts	2.5 g/day PO	Obesity human	Assay of TG,LDL-C, non-HDL-C, TC/HDL-C and LDL-C/HDL-C	Anti-obesity	[49]
Mouse	Anthocyanin-rich BSB seed coat	60 mg/kg/PO	Collagen induced arthritis	Assay of histological inflammation, cartilage scores, oxidative stress markers, pro-inflammatory cytokines and NF-κB signaling	Anti-arthritic activity	[50]
Apc (Min/+) mice	Anthocyanin-rich BSB seed coat	0.2% or 0.5% /kg/PO	-	Assay of Number of intestinal tumors, and cellular expression of β-catenin	Anti-cancer activity	[51]
Apc(Min) mouse	Cyanidin-3-glucoside	0.03%, 0.1% or 0.3%	-	Assay of Plasma, urine and intestinal mucosaanthocyaninswere determined by HPLC, UV spectrophotometry and tandem MS	Anti-cancer activity	[52]
Human hepatoma (HepG2) cells	BSB seed coats	67 µg/mL	Hydrogen peroxide	Assay of ERK, intracellular total protein phosphatase activity	Anti-cancer activity	[53]
Human brain neuroblastoma SK-N-SH cells	Cyanidin-3-O-glucoside, Delphinidin-3-O-glucoside, and petunidin-3-O-glucoside	67 µg/mL	Hydrogen peroxide	Assay of cell viability, ROS, expression of heme oxygenase (HO)-1 ,MAP kinase, ASK1-JNK/p38 pathways by MTT assay, DCF-DA assay, RT-PCR, and Western blotting	Anti-cancer activity	[54]
Humanhepatoma (HepG2) cells and ICR mice	BSB seed coats extracts	25 μg/mL	Benzo[a]pyrene	Assay of cytochrome P4501A1 expression, Nrf2 to antioxidant response elements	Anti-cancer activity	[55]
Ratpheochromocytoma (PC12 cell line)	non-anthocyanin fraction	3, 6, 12, and 25 μg/mL	Amyloid β peptide	Assay of cellular oxidative stress by using DCF-DA, MTT, LDH, MDA level, acetylcholinesterase activity	Anti-amnesic effect	[56]
Human lens epithelial cell line (HLE-B3)	BSB seed coats extracts	0, 50, 100 and 200 μg/mL	Hydrogen peroxide	Assay of apoptosis by Annexin V assay and APO-BrdU TUNEL assay; Western blot and immunostaining of apoptosis-related molecules; Bcl2, Bax, p53 and caspase-3.	Anti- cataract effect	[57]
Rat primary cortical neuron cells	BSB (cv. Cheongja 3, Glycine max (L.) MERR.) seed coat	50 mg/mL	Glutamate	Assay of LDH, MTT, Intracellular ROS and immunofluorescence	Neuroprotective effect	[58]
3T3-Ll cells db/db mice	Anthocyanin cyanidin-3-glucoside	60 mg/kg/PO	-	Assay of PPARγ and C/EBPα gene expressions, TNF-α, PGC-1α, SIRT1 and UCP-3	Antiobesity and antidiabetic effects	[59]
3T3-Ll cells *db*/*db* mice	Anthocyanin cyanidin-3-glucoside	12.5 and 50 μg/mL	-	Assay of MTT, expression of the peroxisome proliferator-activated receptor γ and measurement of lipolysis	Antiobesity and antidiabetic effects	[60]
Wistar albino rats	Anthocyanin cyanidin-3-glucoside	Anthocyanins (24 mg/kg) along with and vitC (100 mg/kg)	10% (*v*/*v*) ethanol	Assay of MTT, expression of GABAB1 receptor,Bax/Bcl-2 ratio, release of cytochrome C and activation of caspase-3 and caspase-9	Neuroprotective effect	[61]
Wistar albino rats	Anthocyanin cyanidin-3-glucoside	Anthocyanins (24 mg/kg) along with and vitamin c (100 mg/kg)	10% (*v*/*v*) ethanol	Assay of GABAB1 receptor, cellular levels of proapoptotic proteins such as Bax, activated caspase-3, and cleaved poly (ADP-ribose) polymerase 1 (PARP-1) intracellular free Ca (2+) level and CaMKII	Neuroprotective effect	[62]
Wistar albino rats	Anthocyanin cyanidin-3-glucoside	Anthocyanins (24 mg/kg) along with and vitamin c (100 mg/kg)	10% (*v*/*v*) ethanol	Assay of expression of glutamate receptors, intracellular signaling molecules, and various synaptic, inflammatory, and apoptotic markers	Neuroprotective effect	[63]
Mouse hippocampal cell line (HT22) and primary prenatal rat hippocampal neurons	Anthocyanin cyanidin-3-glucoside	12.5 and 50 μg/mL	Kainic acid	intracellular Ca^2+^ level, ROS, AMPK, Bcl-2, cytochrome-c, and caspase-3	Antioxidant activity	[64]
Human	BSB seed coat	60 mg/kg/PO	STZ	Assay of glycemic control and lipid metabolism parameters	Anti-hyperlipidemic effect	[37]
in vitro (prostate cancer- DU-145 cells) and in vivo (in athymic nude mouse xenograft model)	Anthocyanin	8 mg/kg	-	Assay of MTT, p53, Bax, Bcl, androgen receptor (AR), and prostate specific antigen	Anti-cancer activity	[65]
HT22 cell lines and adult wister male rats	Anthocyanin	0.2 mg/kg	Amyloid beta 1-42	Assay of MTT, mitochondrial membrane potential, intracellular free Ca^2+^ and apoptotic cells (fluoro-jade B and TUNEL),Western blot analyses were performed	Neuroprotective effect	[66]
Wistar albino rats	Anthocyanin	50 mg/kg/PO	Human fibrin and thrombin solutions	Assay of Masson trichrome and transforming growth factor	Anti-inflammatory and antifibrosis effects	[67]
In vitro	BSB seed coat	388 mg/100 g	-	Assay of DPPH and ABTS+	Antioxidant properties	[68]
Wistar albino rats and rat pheochromocytoma PC12 cell line	Non-anthocyanins	10, 20 mg/kg/PO	H_2_O_2_ and trimethyltin	Assay of MTT, LDH, AChE in vitro inhibition, Y-maze test, Passive avoidance test and MDA levels	Beneficial for neurodegenerative disorders	[69]
Sprague-Dawley rats	BSB	10, 20 mg/kg PO	ciprofloxacin,	Assay of prostate tissue, urine culture, and histological analysis	Anti-inflammatory and antimicrobial effects	[70]
In vitro	Black soybean tea	10, 20 mg/kg PO	-	Assay of DPPH, ferrous ion chelating ability and reducing power	Antioxidant activity	[71]
In vitro	*Aspergillus awamori-*fermented BSB	10, 20 mg/kg PO	-	Assay of DPPH, ferrous ion chelating ability and reducing power	Antioxidant activity	[72]
In vitro	20 soybean hybrids	10, 20 mg/kg PO	-	Assay of DPPH	Antioxidant activity	[73]
In vitro	BSB hybrids	10, 20 mg/kg PO	-	Assay of DPPH, ferric reducing antioxidant power, oxygen radical absorbance capacity	Antioxidant activity	[34]
In vitro	BSB hybrids	10, 20 mg/kg PO	-	Assay of total phenolic content, total flavonoid content, condensed tannin content, monomeric anthocyanin content, DPPH free radical scavenging activity, ferric reducing antioxidant power, and oxygen radical absorbing capacity	Antioxidant activity	[74]
In vitro	30 BSB hybrids	10, 20 mg/kg PO	-	Assay of total phenolic content, total flavonoid content, condensed tannin content, monomeric anthocyanin content, DPPH free radical scavenging activity, ferric reducing antioxidant power, and oxygen radical absorbing capacity	Antioxidant activity	[75]
Male Sprague-Dawley rats	Anthocyanins	6 mg/kg and 24 mg/kg PO	-	Assay of body weight and daily food intake, neuropeptide Y, GABAB1 receptor, protein kinase A-α, and phosphorylated cAMP-response element binding protein	Hypolipidemic and anti-obesity effects	[76]
Wistar albino rats	BSB seed coats	0.037%/PO	High fat diet—16% lard oil	Assay of body weight, adipose tissue weight, and serum lipids	Anti-obesity effect	[77]
C57BL/6 mice	*Monascus pilosus*-fermented BSB	0.5 and 1.0 g/kg/PO	High fat diet—16% lard oil	Assay of blood glucose, TC, leptin and measurement of epididymal, retroperitoneal, and perirenal fat pads	Anti-obesity effect	[78]
Male KK-*A^y^*diabetic mice and L6 myotubes	BSB seed coat extract	22.0 g of BE/kg diet/PO	-	Assay of blood glucose, insulin, AMP-activated protein kinase, glucose transporter 4	Anti-diabetic effects	[79]
Gastric adenocarcinoma, ATCC CRL 1739	Anthocyanin	50 µg/mL	-	Assay of cell viability, ROS, Western blot analyses, RT-PCR were performed to assess gene and protein expression	Anti-oxidative, antibacterial and anti-inflammatory effects	[80]
Immortalized epidermal keratinocyte cell line (HaCaT) and human neonatal dermal fibroblasts	Anthocyanin	50 µg/mL	H_2_O_2_	Assay of tissue VEGF, TSP1, CD31, NF-κB, and phosphorylation of IκBα	Wound healing properties	[81]
Human dermal fibroblasts and keratinocytes cell lines	BSB seed coat extracts	100 µg/mL	-	Assay of TNF-alpha, NF-kB, p65, VEGF in in fibroblasts and keratinocytes	Anti-inflammatory effects	[82]
Wistar albino rats	BSB seed coat extracts	50 and 100 mg/kg/PO	-	Assay of TNF-alpha, ICAM, NF-kB,cyclooxygenase-2, VEGF in in fibroblasts and keratinocytes	Anti-inflammatory properties against ischemia-reperfusion injury	[83]
Bovine aortic endothelial cells and male Sprague-Dawley rats	Anthocyanin BSB seed coat	25, 50 and 100 mg/kg/PO	LAD occlusion and reperfusion	Assay of MTT, Luciferase, TNF-alpha, ICAM, NF-kB, cyclooxygenase-2, vascular endothelial growth factor	Cardioprotective effect	[84]
Murine BV2 microglial cells	Anthocyanin BSB seed coat	100 µg/mL	Lipopolysaccharides	Assay of NO, prostaglandin E(2), and pro-inflammatory cytokines, including TNF-α IL-1β, NO synthase, cyclooxygenase-2, NF-κB, ERK, c-JNK, p38 MAP kinase, and Akt.	Anti-inflammatory and potent neurodegenerative diseases	[85]
U2OS cells	Anthocyanin BSB seed coat	200 μg/mL	-	Assay of extracellular signal-regulated kinase 1/2, p38 mitogen-activated protein kinase, c-Jun N-terminal kinase, protein kinase B and adenosyl mono-phosphate-dependent protein kinase	Anticancer effects	[86]
Wistar albino rats	Anthocyanin	40 or 80 mg/kg PO	Varicocele-induced rats	Histological examination and semen analysis	Anti-infertility effects	[87]
Wistar albino rats	Anthocyanin	40, 80, and 160 mg/kg PO	benign prostatic hyperplasia-induced rats	Assay of apoptosis in the prostates by the TUNEL assay	Anti-infertility effects	[88]
Wistar albino rats	Anthocyanin BSB seed coat	50 mg/kg PO	*N*-methyl-*N*-nitrosourea	Electro-retinographic recordings and morphological analyses	Anti-blindness	[89]
Detroit 551 cells	fermented BSB broth	200 μg/mL	-	Assay of DPPH radical scavenging effect, reducing power and ferrous ion chelating effect.	Antioxidant effect	[90]
Human U87 glioma cells	Anthocyanin BSB seed coat	100 μg/mL	-	Assay of autophagy, Atg5 expression	Anti- stroke effect	[91]
Wistar albino rats	citric acid fermented of BSB	10 mL/kg	Ferricnitrilotriacetate	Assay of antioxidative enzymes including catalase, glutathione peroxidase, glutathione reductase, glutathione *S*-transferase, glucose-6-phosphate dehydrogenase, quinone reductase, serum creatinine and urea nitrogen	Anti- renal tubular oxidative damage	[92]
In vitro	BSB fermented with either *Bacillus subtilis* BCRC 14715 or *Bacillus* sp. CN11	2, 4, 6 mL	-	Assay of ACE inhibitory activity and the reducing power of the fermented BSB	Antioxidant activity	[93]
In vitro	solid fermentation of steamed BSB	100 μg/mL	4-nitroquinoline-*N*-oxide and Benzo[a]pyrene	Assay of mutagenicity	Mutagenicity and antimutagenicity effects	[94]
In vitro	BSB with *Bacillus subtilis* BCRC 14715	100 μg/mL	water, 80% methanol, 80% ethanol, 80% acetone	Assay of DPPH radical-scavenging effect, and Fe^2+^-chelating activity	Antioxidant activity	[95]
Wistar albino rats and In vitro	Anthocyanin BSB seed coat	100 μg/mL	UVB-induced apoptotic cell death	Assay of caspase-3, Bax, NF-κB, cylooxygenase-2	Anti-skin cancer	[96,97]
In vitro	Hot water extracts of BSB	100 μg/mL	Human adenovirus type 1 and coxsackievirus B1	WST assay and in vitro antiviral assay	Antiviral activity	[98]

ABTS—2,2′-azino-bis(3-ethylbenzothiazoline-6-sulphonic acid; AChE—acetyl choline esterase; ASK—1-Apoptosis signal-regulating kinase 1; BSB—Black soybeans; cAMP—cyclic adenosine mono phosphate; CD—cluster of differentiation; DCF—DA-dichlorofluorescein diacetate; DNA—Deoxy ribonucleic acid; DPPH—2,2-diphenyl-1-picrylhydrazyl; ERK—extracellular signal–regulated kinases; GABA—γ-aminobutyric acid; GFAP—Glial fibrillary acidic protein; HDL—C-high density lipoprotein cholesterol; HO—heme oxygenase; HPLC—high performance liquid chromatography; ICAM—intracellular adhesion molecule-1; IL—Interleukin; JNK—Jun N-terminal Kinase; LDH—lactate dehydrogenase; LDL-C—LDL-cholesterol; MAP kinase—mitogen-activated protein kinase; MDA—melondialdehyde; MS—mass spectroscopy; MTT—3-(4,5-dimethylthiazol-2-yl)-2,5-diphenyltetrazolium bromide; NF-_k_B—nuclear factor kappa B; NO—nitric oxide; NRF-1—Nuclear factor erythroid 2-related factor 1; PARP—Poly (ADP-ribose) polymerase; PPAR—peroxisome proliferator-activated receptors; ROS—reactive oxygen species; RT-PCR—reverse transcriptase-polymerase chain reaction; TC—total cholesterol; TG—Triglycerides; TNF—tumor necrosis factor; TSP1—thrombospondin; VCAM—vascular cell adhesion molecule-1; VEGF—Vascular endothelial growth factor; PO—per oral.
